# Green Synthesis of Silver Nanoparticles Using *Pseudoduganella eburnea* MAHUQ-39 and Their Antimicrobial Mechanisms Investigation against Drug Resistant Human Pathogens

**DOI:** 10.3390/ijms21041510

**Published:** 2020-02-22

**Authors:** Md. Amdadul Huq

**Affiliations:** Department of Food and Nutrition, College of Biotechnology and Natural Resource, Chung-Ang University, Anseong-si, Gyeonggi-do 17546, Korea; amdadbge100@cau.ac.kr

**Keywords:** green synthesis, AgNPs, *Pseudoduganella eburnea* MAHUQ-39, antibacterial mechanisms

## Abstract

Silver nanoparticles (AgNPs) have shown great promise in biomedical applications. The exact mechanism and mode of action of AgNPs regarding antimicrobial activity are still not well known. Moreover, synthesis of nanoparticles by physical and chemical methods is expensive and not ecofriendly. This study highlights the green, rapid, facile, cost-effective and ecofriendly synthesis of AgNPs using *Pseudoduganella eburnea* MAHUQ-39 and also investigates their antibacterial mechanisms. The transmission electron microscopy (TEM) image revealed a spherical shape of the AgNPs. The size of the synthesized AgNPs was 8 to 24 nm. The elemental mapping and selected area electron diffraction (SAED) and X-ray diffraction (XRD) patterns revealed the crystalline structure of AgNPs. Fourier-transform infrared spectroscopy (FTIR) analysis identified the functional groups that are involved in the reduction of silver ion to AgNPs. The green synthesized AgNPs exhibited strong antimicrobial activity against multidrug-resistant pathogenic microbes. Minimal inhibitory concentrations (MICs) of *Staphylococcus aureus* and *Pseudomonas aeruginosa* were 100 μg/mL and 6.25 μg/mL, respectively, and the minimum bactericidal concentrations (MBCs) of *S. aureus* and *P. aeruginosa* were 200 μg/mL and 50 μg/mL, respectively. Our data demonstrated that synthesized AgNPs created structural changes of cells and destroyed the membrane integrity of strains *S. aureus* and *P. aeruginosa*. Therefore, AgNPs synthesized by strain MAHUQ-39 can be used as a powerful antimicrobial agent for various therapeutic applications.

## 1. Introduction

Nanoparticles (NPs) are a wide class of materials that attract many researchers because of their small size (1–100 nm), unique properties, large surface area, increased reactivity, ability to easily enter the human body and different applications in various fields of modern science, including agricultural and medical sciences. Different kinds of nanoparticles have been reported, including zinc, gold, silver, copper, alginate, titanium and magnesium [1,2,3]. Silver nanoparticles (AgNPs) are broadly used nanoparticles because of their potential applications in various fields of science, including optoelectronics, optics, pharmaceutical and medical sciences for the development of drug delivery systems, therapeutic agents, biosensors, gene therapy, etc. [4,5,6,7]. AgNPs also have been examined for remarkable applications such as on patients for bone cement, trauma, prostheses, dental implants, protective clothing [8,9], etc.

It is reported that AgNPs have strong antimicrobial, antibiofilm, anti-inflammatory and anticancer activities, out of which the antimicrobial properties of AgNPs protect against pathogenic microorganisms, including viruses, bacteria and eukaryotic micro-organisms, have been exploited [3,10]. Previous reports suggest that AgNPs have strong sterilization and bactericidal effect against many drug-resistant pathogenic microorganisms [11]. Because of the effective antimicrobial properties of AgNPs, use of them has been commercialized in various industrial products, such as for footwear, wound dressings, paints, hand gel, medical catheter coverings, plastics, cosmetics, etc. [12]. The exact mechanism and mode of action behind the antimicrobial activity of AgNPs are still not well known. Despite that, there are some proposed mechanisms for the mode of action of AgNPs, such as disturbance of the cell membrane or cell wall, by damaging DNA and proteins or alteration of nutrient uptake and electron transport, which lead to the death of cells [13,14,15]. AgNPs are becoming an ideal candidate in biological and medical sectors due to their antimicrobial, antibiofilm, anti-inflammatory, anticoagulant and anticancer efficacies [3].

Physical and chemical methods are widely used for the synthesis of nanoparticles, but unfortunately, these methods have some drawbacks such as their use of high energy or hazardous chemicals, great expense and production of large amounts of toxic byproducts that create environmental contamination [16]. To overcome these limitations of physical and chemical methods, ecofriendly, facile, low cost and nontoxic methodologies are essential for metal nanoparticle synthesis by avoiding the use of toxic and expensive chemicals and solvents [7]. Recently, biological synthesis of metal nanoparticles has been highlighted, which is safe, ecofriendly, facile, nontoxic and economical [3]. For biosynthesis of metal nanoparticles, bacteria have received the most attention due to their growing success, handling ease, manipulation and genetic modification [17]. In recent times, several microorganisms, such as *Bacillus methylotrophicus*, *Brevibacterium frigoritolerans* and *Novosphingobium* sp. THG-C3 have been isolated for green synthesis of silver nanoparticles [7,15,18]. Soil is a good source of microbial diversity and these diverse bacteria can be used for human welfare. Soil bacteria also need inexpensive and less nutrient media for growth that make them a perfect candidate for their utilization. In this study, bacterial strain *Pseudoduganella eburnea* MAHUQ-39 was isolated from a soil sample and used for the facile, rapid and ecofriendly synthesis of AgNPs. Bacteria secrete various enzymes in culture supernatant, including reductases that are responsible for the biosynthesis of nanoparticles [19]. In this study, culture supernatant of *Pseudoduganella eburnea* MAHUQ-39 was used for the rapid, facile and green synthesis of AgNPs without adding any reducing agent. Moreover, the antibacterial activity of green synthesized AgNPs was investigated against multidrug resistant human pathogens.

## 2. Results and Discussion

### 2.1. Characterization of AgNPs Producing Bacterial Strain

**Figure 1:**
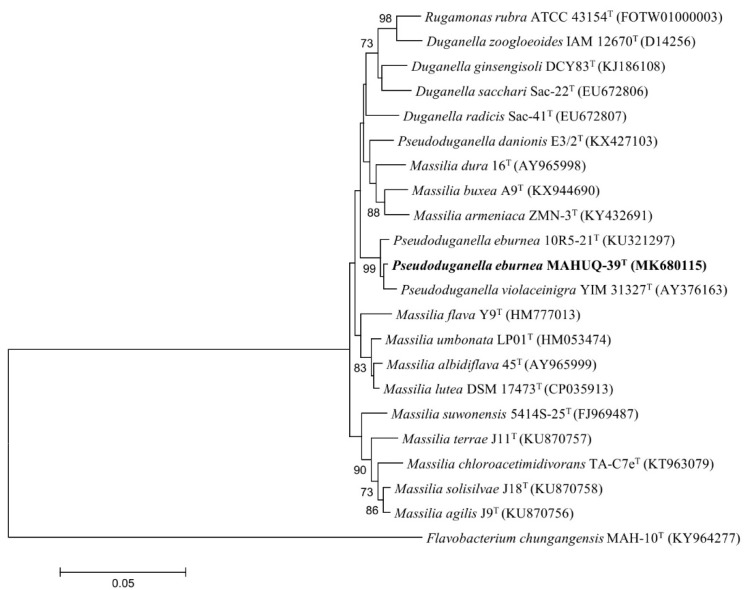
The neighbor-joining (NJ) tree on the basis of 16S rRNA gene sequences analysis demonstrating the phylogenetic relationships of isolated strain MAHUQ-39 with related type strains. Bootstrap values more than 70% are shown at branching points. The scale bar shows 0.05 substitutions per nucleotide position.

The 16S rRNA gene sequence of strain MAHUQ-39 was 1412 bp and the sequence was submitted to NCBI (accession number MK680115). On the basis of the 16S rRNA gene sequence analysis, strain MAHUQ-39 showed the highest sequence similarity with *Pseudoduganella eburnea* 10R5-21^T^ (99.5%). The phylogenetic analysis using a neighbor-joining tree also indicated that strain MAHUQ-39 was affiliated with the genus *Pseudoduganella* (Figure 1). The biochemical characterization of isolated strain MAHUQ-39 was carried out using API 20 NE and API ZYM; the results are shown in Table 1.

### 2.2. Green Synthesis of AgNPs Using Strain MAHUQ-39

**Figure 2:**
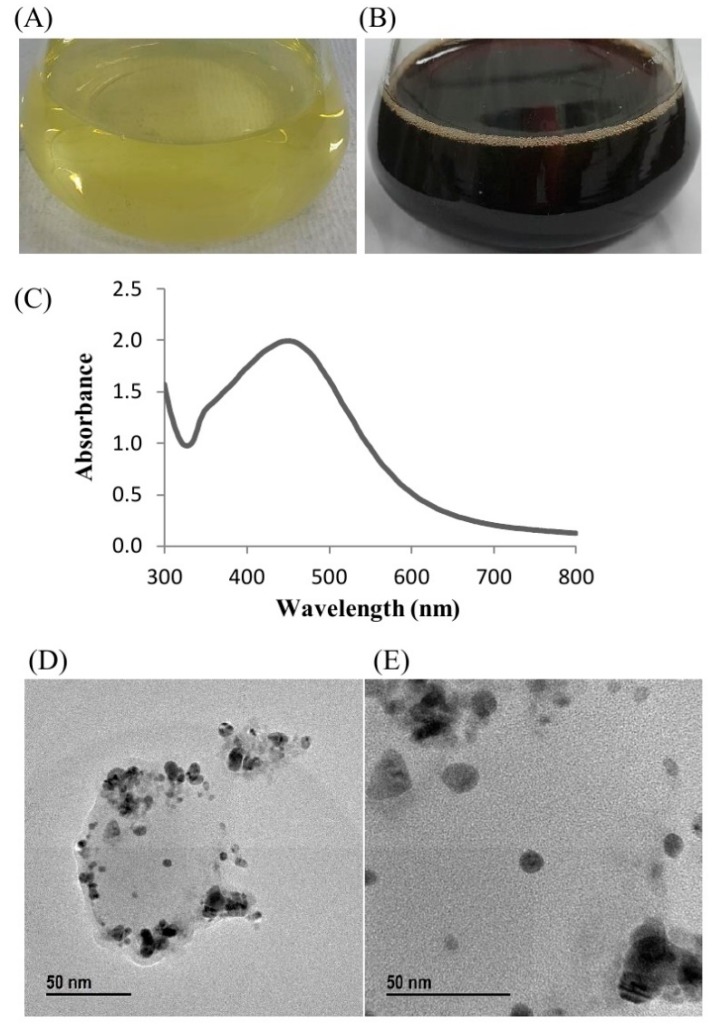
R2A broth with AgNO_3_ as control (**A**), synthesized silver nanoparticles (AgNPs) (**B**), UV–vis spectra (**C**) and field emission–transmission electron microscopy (FE–TEM) images of synthesized silver nanoparticles (**D**,**E**).

Green synthesis of AgNPs using *Pseudoduganella eburnea* MAHUQ-39 was confirmed by observing the color of the culture supernatant. The color of MAHUQ-39 culture supernatant changed to deep brown from watery yellow within 24 h of incubation (Figure 2B), which reveals the synthesis of AgNPs, as the biosynthesized silver nanoparticles cause surface plasmon resonance [3]. Control (without bacteria) did not show any change in color when incubated for the same conditions and period (Figure 2A). The optimum temperature (30 °C) and AgNO_3_ concentration (1 mM final concentration) for the stable synthesis of AgNPs were determined on the basis of ultraviolet–visible (UV–vis) spectral analysis (Supplementary Figures S1 and S2). The accurate mechanism of synthesis of AgNPs using bacteria is still at an initial stage. Previous reports suggest that various enzymes secreted by bacteria are an important part of the green synthesis of nanoparticles. First, the metal ions are trapped on the surface or inside of bacterial cells and then the trapped metal ions are converted to nanoparticles in the presence of bacterial enzymes [20,21]. Two methods are available for the biosynthesis of silver nanoparticles using microorganisms which can be classified as intracellular or extracellular, depending on the place of nanoparticle synthesis. Extracellular synthesis of nanoparticles is more convenient, facile and cost-effective as the synthesized nanoparticles can be easily and quickly purified, in contrast to intracellular synthesis, which needs difficult and complex purification steps. In the present study, the extracellular methodology was used to green synthesize AgNPs using *Pseudoduganella eburnea* MAHUQ-39, which is simple, facile, cost-effective and ecofriendly.

### 2.3. Characterization of Green Synthesized AgNPs

**Figure 3:**
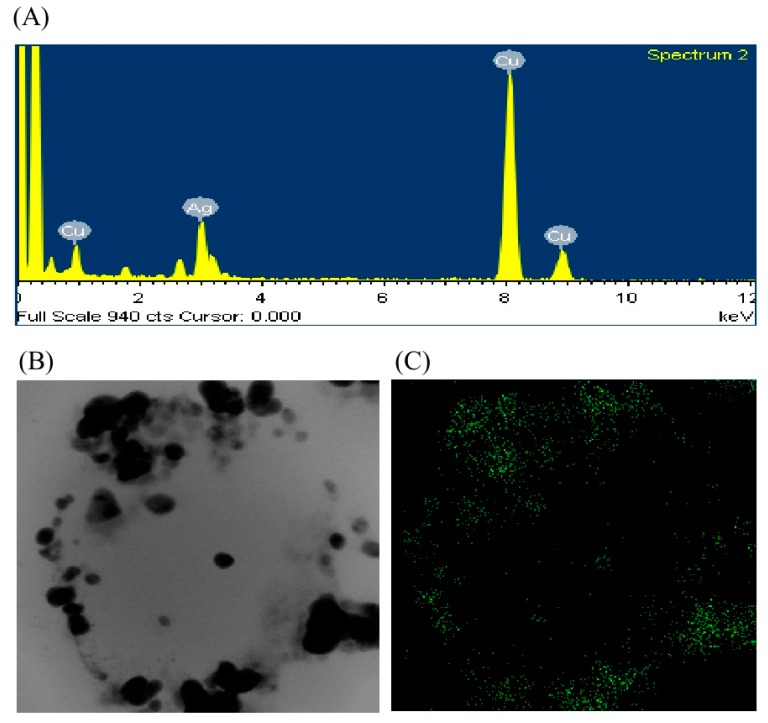
Energy dispersive X-ray (EDX) spectrum of synthesized AgNPs (**A**), Field emission–transmission electron microscopy (FE–TEM) image used for elemental mapping (**B**) and distribution of silver in elemental mapping (**C**).

**Figure 4:**
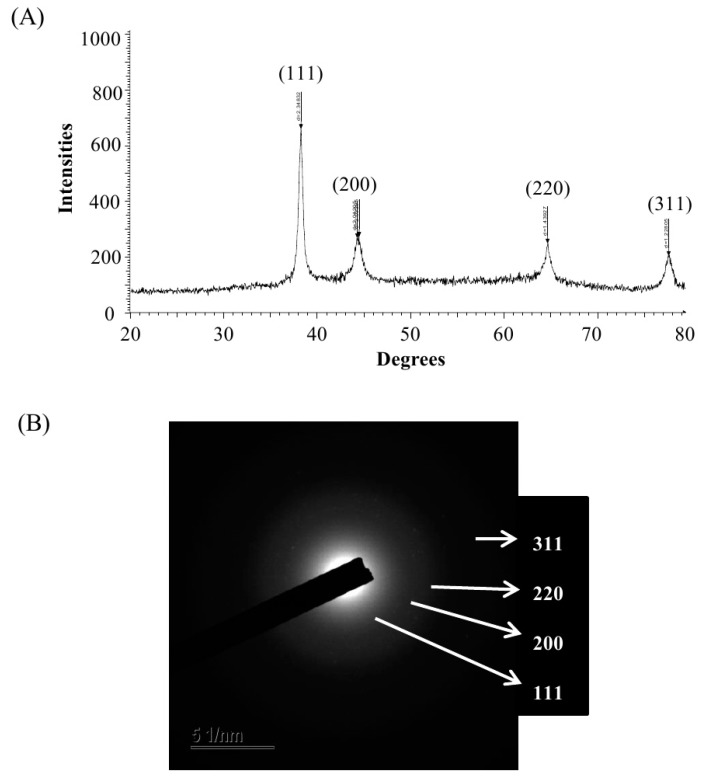
X-ray diffraction pattern (**A**) and elemental mapping and selected area diffraction (SAED) pattern (**B**) of synthesized AgNPs.

The biosynthesis of AgNPs was assured by UV–vis spectral scanning in a range of 300–800 nm; a strong peak at 448 nm was observed (Figure 2C). It is reported that the absorption peak at about 400–450 nm is due to surface plasmon resonance of AgNPs, which confirmed the formation of AgNPs [22]. Field emission–transmission electron microscopy (FE–TEM) images showed clear morphology of green synthesized AgNPs. FE–TEM analysis disclosed a spherical shape of synthesized AgNPs with a size range of 8 to 24 nm (Figure 2D,E). Previous studies also showed a similar size and shape of biosynthesized AgNPs [15]. The composition and purity of green synthesized AgNPs were analyzed using an EDX spectrometer. The EDX spectrum exhibited elemental signals of silver atoms in biosynthesized AgNPs at around 3 keV and revealed homogenous distribution of AgNPs (Figure 3A). Previous reports also suggest that the EDX pattern of AgNPs commonly exhibits an absorption peak nearly at 3 keV [18]. The EDX spectrum also showed other signals for copper and carbon due to the use of a copper grid in the FE–TEM. The highest distribution of silver elements in the sample was found through the elemental mapping of green synthesized AgNPs (Figure 3B,C). The XRD spectrum of the synthesized AgNPs revealed the diffraction peaks at 2θ values of 38.13°, 44.31°, 64.44° and 77.42°, matching the corresponding 111, 200, 220 and 311 lattice planes of AgNPs. These diffraction peaks may have resulted from the capping agents that are responsible for the stabilization of nanoparticles. The absence of other peaks reveals the purity of green synthesized AgNPs (Figure 4A). Similar XRD results were found in previous studies that showed the biosynthesis of silver nanoparticles using microorganisms [3,15]. The elemental mapping and selected area diffraction (SAED) pattern exhibited sharp rings that indicate the crystalline structure of green synthesized AgNPs and correspond to the reflections at 111, 200, 220 and 311 (Figure 4B). The crystalline structure of biosynthesized AgNPs was confirmed from both the SAED pattern and XRD spectrum.

**Figure 5:**
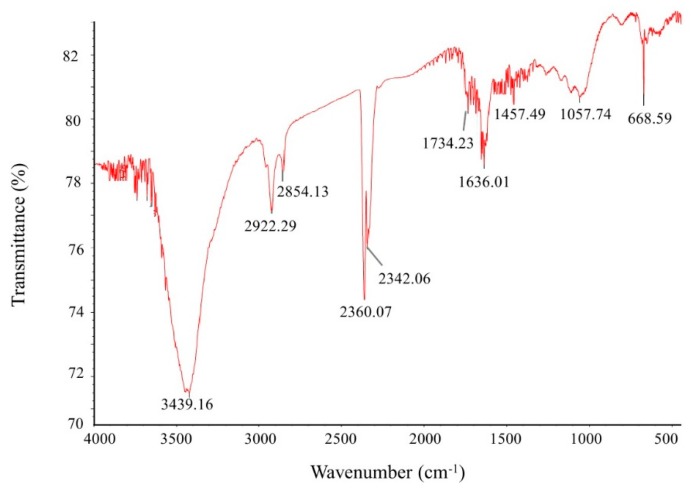
Fourier-transform infrared spectroscopy (FTIR) spectra of synthesized silver nanoparticles.

**Figure 6:**
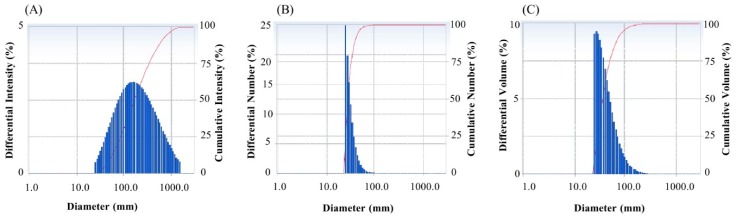
Particle size distribution of synthesized AgNPs according to intensity (**A**), number (**B**) and volume (**C**).

Fourier-transform infrared spectroscopy (FTIR) is an important tool for the observation of functional groups which are involved for the stabilization of synthesized nanoparticles. FTIR spectrum of green synthesized AgNPs reveals clear peaks throughout the whole range of observation. FTIR analysis expressed visible bands at 3439.16, 2922.29, 2854.13, 2360.07, 2342.06 1734.23, 1636.01, 1457.49, and 1057.74 cm^−1^ for synthesized AgNPs (Figure 5). The band found at 3439.16 cm^−1^ can be assigned to O–H (alcohol) and/or N–H (amine) stretching. The bands seen at 2922.29 and 2854.13 cm^−1^ are attributed to C–H (alkane) stretching. The peaks observed at 2360.07 and 2342.06 cm^−1^ correspond to the O=C=O (carbonyl bond group) stretching. The peak seen at 1636.01 cm^−1^ represents the N–H (amine) bend. The bands seen at 1457.49 cm^−1^ and 1057.74 cm^−1^ represent the C–H (alkane) bend and C–O (alcohol/ether) stretching, respectively. The FTIR data indicate that the biological molecules could possibly be involve for both synthesis and stabilization of AgNPs. The present data were supported by the previous report, which showed the microbial synthesis of nanoparticles [23]. The particle size of green synthesized AgNPs was calculated by dynamic light scattering (DLS) analysis. Figure 6 shows the particle size distribution of biosynthesized AgNPs on the basis of volume, number and intensity. The average particle size of synthesized AgNPs was about 141.2 nm with a polydispersity value of 0.369. The polydispersity value indicates the good quality of synthesized AgNPs. Previous reports also showed similar nanoparticle size when synthesized by microorganisms [3,7].

### 2.4. Antimicrobial Activity of Green Synthesized AgNPs

**Figure 7:**
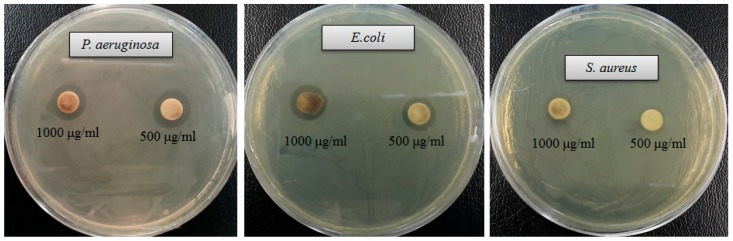
Zones of inhibition of synthesized AgNPs (0.03 mL) at 500 μg/mL and 1000 μg/mL concentrations in water against *Pseudomonas aeruginosa, Escherichia coli* and *Staphylococcus aureus*.

**Figure 8:**
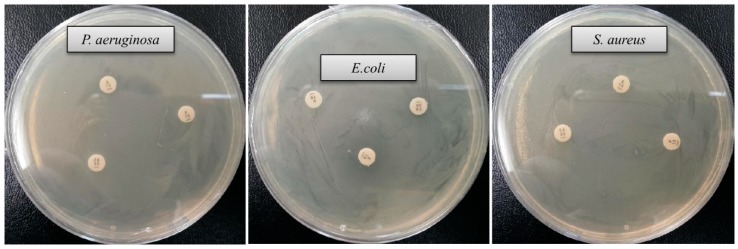
Antibacterial activity of commercial antibiotics against *P. aeruginosa*, *E.coli* and *S. aureus*. Abbreviations: P (penicillin G, 6 μg/disc), E (erythromycin, 15 μg/disc) and VA (vancomycin, 30 μg/disc).

Drug-resistant pathogenic bacteria have become a major concern for human health, as it is difficult to control these pathogens by using currently available antibiotics. Green synthesized AgNPs can be used to overcome the drawbacks of commercial antibiotics. Biosynthesized AgNPs are well known for their antimicrobial potency against various pathogenic microorganisms [3,15,17,24]. In the current study, AgNPs were synthesized using a biological process and their antibacterial potency was investigated against drug-resistant pathogenic microbes (*Staphylococcus aureus*, *Escherichia coli* and *Pseudomonas aeruginosa*). Results showed that synthesized AgNPs have significant antimicrobial activity against all tested human pathogens such as *S. aureus*, *P. aeruginosa* and *E. coli* (Figure 7). Figure 7 reveals the clear zone of inhibition around the discs saturated with synthesized AgNPs (0.03 mL/disc), at both 500 μg/mL and 1000 μg/mL. The antibacterial efficacy against pathogenic microorganisms was measured by calculating the diameter of the zone of inhibition (Table 2). The synthesized AgNPs exhibited the highest antibacterial activity against drug resistant *P. aeruginosa* followed by *E. coli* and *S. aureus*. The results of this study propose that the green synthesized AgNPs were able to control the multidrug resistant pathogenic bacteria and could be used in the medical field. Commercial antibiotics such as vancomycin, erythromycin and penicillin G were also used to check their antibacterial efficacy against human pathogens such as *P. aeruginosa*, *E. coli* and *S. aureus*. All three human pathogens were resistant to the commercial antibiotics vancomycin, erythromycin and penicillin G, and did not show any inhibition zone around the antibiotic discs (Figure 8, Table 3).

### 2.5. Minimum Inhibitory Concentration and Minimum Bactericidal Concentration

**Figure 9:**
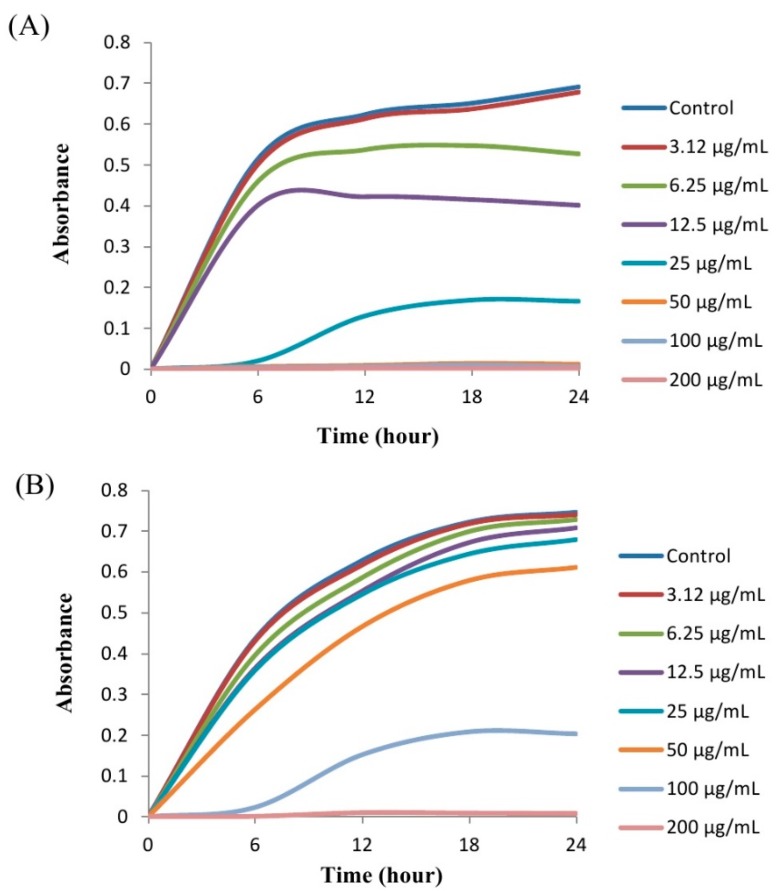
Growth curves of *P. aeruginosa* (**A**) and *S. aureus* (**B**) cultured in R2A broth with various concentrations of synthesized AgNPs.

**Figure 10:**
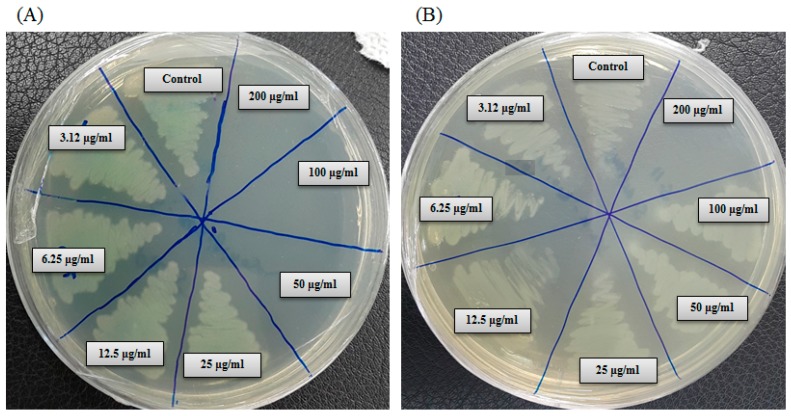
Minimum bactericidal concentration (MBC) of synthesized AgNPs against *P. aeruginosa* (**A**) and *S. aureus* (**B**).

In the current study, the minimum inhibitory concentration (MIC) and minimum bactericidal concentration (MBC) of AgNPs against *P. aeruginosa* and *S. aureus* were determined by a standard broth dilution assay. Bacterial growth curves exhibiting the MICs of green synthesized AgNPs were 6.25 μg/mL and 100 μg/mL for *P. aeruginosa* and *S. aureus*, respectively, indicating that the synthesized AgNPs strongly suppressed the proliferation of these two pathogens (Figure 9). The MICs of the AgNPs synthesized by strain MAHUQ-39 were much lower than other antimicrobial agents, including biosynthesized ZnO NPs, which were 8 mg/mL and 4 mg/mL against *E. coli* and *S. aureus*, respectively [25]. The MBCs of biosynthesized AgNPs for *P. aeruginosa* and *S. aureus* were 50 μg/mL and 200 μg/mL, respectively (Figure 10), which were also much lower than those of other antimicrobial agents [25]. The MIC and MBC of green synthesized AgNPs were higher for *S. aureus* than those for *P. aeruginosa*. The current and previous data displayed a higher AgNPs susceptibility of Gram-negative bacteria compared to that of Gram-positive bacteria. This might due to distinctions in the composition of their cell wall [26].

### 2.6. Study of Morphogenesis of Treated Cells by FE–SEM

**Figure 11:**
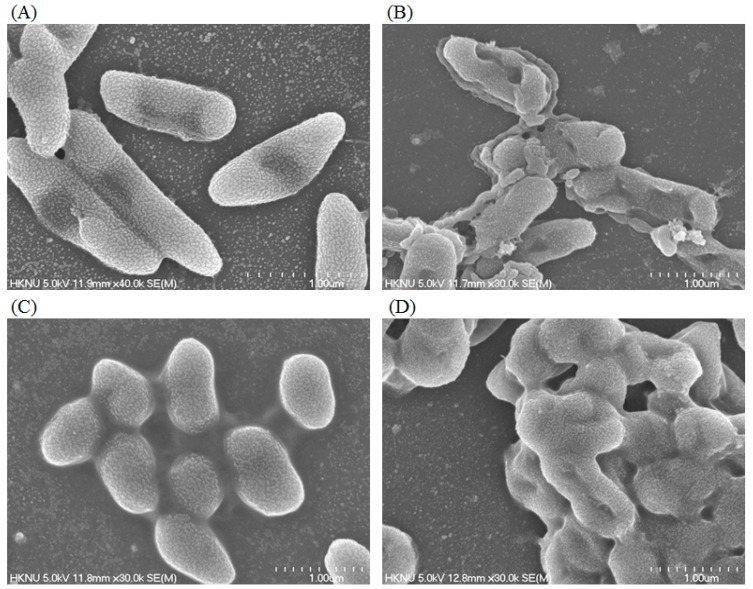
FE–SEM images of normal *P. aeruginosa* cells (**A**), 1 × MBC AgNPs treated *P. aeruginosa* cells (**B**), normal *S. aureus* cells (**C**), 1 × MBC AgNPs treated *S. aureus* cells (**D**).

Morphological and ultrastructural changes of *P. aeruginosa* and *S. aureus* cells treated with green synthesized AgNPs were directly observed by FE–SEM (Figure 11). By examining the FE–SEM, the untreated *P. aeruginosa* cells were seen as typically normal rod-shaped and intact cell surfaces with no damage (Figure 11A). However, *P. aeruginosa* cells treated with 1 × MBC of synthesized AgNPs displayed irregularly wrinkled, damaged, deformed and cracked outer surfaces with collapsed cell membranes (Figure 11B). Similarly, untreated *S. aureus* cells were also seen as regular plump and sphere-shaped, with intact and smooth cell surfaces (Figure 11C), while the cells treated with 1 × MBC of biosynthesized AgNPs were deformed and damaged in the middle of the cell. The normal shape of *S. aureus* cells was changed and appeared as distorted and damaged (Figure 11D). The morphological changes and damages of bacterial cell walls revealed that the AgNPs might interfere with the normal function of cells. The images revealed a higher sensitivity of *P. aeruginosa* than *S. aureus*, which might due to the Gram-negative bacteria and Gram-positive bacteria, as they have a cell wall with different structures and components. Similar results were shown by Chatterjee et al., who investigated the cell damage mechanism of silver nanoparticles against *E. coli* and *S. aureus* [26]. There are some mechanisms for the antimicrobial activities of AgNPs that have been proposed and reported. The widely accepted mechanism of antimicrobial activity of AgNPs is that the positively charged silver ions present in AgNPs can interfere with different negatively charged phosphorus or sulfur-bearing bio-macromolecules such as proteins and nucleic acid. This causes structural alterations in bacterial cells, deformations and damages of the cell wall and cell membrane that leads to interruption of metabolic processes, and finally cell death [27]. Reports also suggest that different free radicals that are derived from the surface of nanoparticles are responsible for the damage to the cell membrane by enhancing membrane permeability, which leads to cell death [28].

## 3. Materials and Methods 

### 3.1. Materials

All media were bought from Difco, MB Cell (Seoul, Korea). Standard antibiotics discs of erythromycin (E15) 15 μg/disc, penicillin G (P10) 6 μg/disc and vancomycin (VA30) 30 μg/disc were purchased from Oxoid Ltd., England. Analytical grade AgNO_3_ (silver nitrate) was obtained from Sigma–Aldrich (St. Louis, MO, USA). The pathogenic bacterial strains *Pseudomonas aeruginosa* [ATCC 10145], *Escherichia coli* [ATCC 10798], and *Staphylococcus aureus* [ATCC 6538] were obtained from the American Type Culture Collection (ATCC).

### 3.2. Isolation and Identification of Strain MAHUQ-39

The bacterial strain *Pseudoduganella eburnea* MAHUQ-39 was isolated from a soil sample collected from a pear orchard, located in Anseong, Republic of Korea. One gm of soil sample was dissolved in 10 mL 0.85% NaCl sterile NaCl solution, serially diluted and spread on R2A agar (Reasoner’s 2A agar) plates containing 0.5 gm proteose peptone No. 3, 0.5 gm yeast extract, 0.5 gm casamino acids, 0.5 gm soluble starch, 0.5 gm dextrose, 0.3 gm dipotassium phosphate, 0.3 gm sodium pyruvate, 0.05 gm magnesium sulfate and 15 gm agar in 1000 mL distill water. To check the AgNPs synthesis ability, the isolated strains were streaked on an R2A agar plate supplemented with 1 mM filter-sterilized AgNO_3_ solution. The plates were incubated in an incubator for 48 h at 30 °C and then investigated for bacterial growth. The positive strain was preserved at −80 °C as a suspension in R2A broth with 25% glycerol (*v*/*v*). Strain MAHUQ-39 has been deposited in the Korean Agricultural Culture Collection (KACC number 21243). Genomic DNA of isolated strain MAHUQ-39 was extracted using bacterial a Genomic DNA extraction kit (Solgent, Korea). The 16S rRNA gene was amplified using bacterial universal primers (27F and 1492R) and sequenced by Solgent Co., Ltd. (Daejeon, Korea). The 16S rRNA gene sequences of related type strains were collected from the EzTaxon–e server and GenBank database [29]. To discover the phylogenetic location of strain MAHUQ-39, its phylogenetic tree was constructed using the neighbor-joining method [30] and the MEGA6 program package [31]. Enzyme activities and biochemical characteristics of strain MAHUQ-39 were examined using API 20NE and API ZYM kits (bioMe’rieux, France) according to the manufacturer’s instructions.

### 3.3. Green Synthesis of AgNPs Using Strain MAHUQ-39

For the green synthesis of AgNPs, strain *Pseudoduganella eburnea* MAHUQ-39 was cultured in 100 mL of R2A broth and incubated in a shaking incubator with 140 rpm for two days at 30 °C. After two days of incubation, the culture supernatant was collected using a centrifuge at 9000 rpm for 15 min. A 0.1 mL (1 M concentration) filter-sterilized AgNO_3_ solution was added to 100 mL of supernatant and again incubated in an orbital shaker for 24 h at 140 rpm and 30 °C. Synthesis of AgNPs was confirmed by visually observing the changing color of the culture medium. Finally, the biosynthesized AgNPs were collected by high speed centrifugation at 14,000 rpm for 20 min. The pellets of green synthesized AgNPs were washed with distilled water and then air-dried and finally used for characterization and antimicrobial studies.

### 3.4. Characterization of Green Synthesized AgNPs

The green synthesis of AgNPs was monitored and recorded using a UV–vis (ultraviolet–visible) spectrophotometer in the range of 300–800 nm. The morphology, elemental compositions and purity of synthesized AgNPs were investigated by FE–TEM (field emission–transmission electron microscopy), energy dispersive X-ray (EDX) spectroscopy and elemental mapping and selected area diffraction (SAED) pattern. A drop of biosynthesized AgNPs solution (dissolved in distilled water) was kept on a carbon-coated copper grid, then dried at room temperature and, finally, transferred to the microscope for analysis. The XRD (X-ray diffraction) analysis was carried out using an X-ray diffractometer (D8 Advance, Bruker, Germany) over the 2θ range of 30–90°, operated with CuKα radiation, at 40 kv, 40 mA and 6°/min scanning rate. Air-dried samples were used for XRD analysis. The FTIR (Fourier-transform–infrared) spectrum shows the biomolecules that are involved for the biosynthesis and stabilization of AgNPs. FTIR analysis was accomplished by using a PerkinElmer Fourier-transform infrared spectrometer with resolution 4 cm^−1^ in the range of 400–4000 cm^−1^. For FTIR analysis, air-dried samples in powder form were used. Particle size of green synthesized AgNPs were determined by dynamic light scattering (DLS) using a Malvern Zetasizer Nano ZS90 at 25 °C and a 12° angle. Pure water was used as a dispersive medium with dielectric constant 78.3, viscosity 0.8878 cP and refractive index 1.3328.

### 3.5. Antimicrobial Activity of Green Synthesized AgNPs

The disc diffusion method was used to investigate the antimicrobial efficacy of green synthesized AgNPs against human pathogens. The tested pathogenic microbes (*Staphylococcus aureus*, *Pseudomonas aeruginosa* and *Escherichia coli*) were grown overnight in MHB Mueller–Hinton (MH) broth. A 0.1 mL sample of overnight growth bacterial culture of each tested pathogen was spread on a Mueller–Hinton (MH) agar plate. The sterile paper discs (8 mm in diameter) containing 0.03 mL of synthesized AgNPs (both 500 μg/mL and 1000 μg/mL, dissolved in distilled water) were placed on the surface of the inoculated MH agar plates. Then, the plates were incubated in an incubator for 24 h at 37 °C. Similarly, the antibacterial activity of commercial antibiotics such as penicillin G (6 μg/disc), erythromycin (15 μg/disc) and vancomycin (30 μg/disc) was tested against *S. aureus*, *P. aeruginosa* and *E. coli*. The inhibition zones were measured after 24 h of incubation. This test was performed in triplets.

### 3.6. Determination of MIC and MBC

*S. aureus* and *P. aeruginosa* were selected for this study as Gram-positive and Gram-negative model strains, respectively. Both strains were grown in MH broth at 37 °C for overnight and then the turbidity was adjusted to nearly 10^6^ CFUs/mL. MICs of the green synthesized AgNPs were calculated using a broth microdilution assay. Control experiments were carried out without AgNPs. Briefly, serial dilutions of green synthesized AgNPs were prepared in sterile R2A broth with different concentrations ranging from 200 μg/mL to 3.12 μg/mL. A 0.1 mL sample of each AgNPs dilution was kept in a 96-well plate, and inoculated with 0.1 mL of the test organism in R2A broth to a final concentration of 5 × 10^5^ CFU/mL, and further incubated in a shaking incubator at 37 °C for 24 h. The MIC was measured as the lowest concentration of AgNPs at which the growth of the bacterium was entirely inhibited after 24 h. The optical density at 600 nm was recorded every 3 h during incubation using an ultraviolet–visible spectrophotometer. MBC was determined by streaking 0.01 mL of each set on an MH agar plate and further incubating at 37 °C for 24 h. The culture plates were watched by the naked eye to determine the lowest concentration (MBC) that blocked bacterial growth [32].

### 3.7. Investigation of Antibacterial Mechanisms via Study of Morphogenesis of Treated Cells

The morphological changes of strains *S. aureus* and *P. aeruginosa* were examined by SEM. Logarithmic growth phase cells in a concentration of 1 × 10^7^ CFU/mL were treated with the green synthesized AgNPs (dissolved in 0.85% NaCl) at a concentration of 1 × MBC for 6 h. In control, bacterial cultures were treated with 0.85% NaCl solution. Phosphate-buffered saline (PBS) was used to wash the treated cells. A 2.5% glutaraldehyde solution was used to fix the cells for 4 h and then washed with PBS several times. Again, the cells were fixed using 1% osmium tetroxide and then washed with PBS buffer. Fixed cells were dehydrated by different concentrations of ethanol (30%, 50%, 70%, 80%, 95% and 100% for 10 min) at room temperature. The resulted samples were dried using a desiccator. Finally, the cells were placed on SEM metallic stubs and coated with gold. The morphological and structural alterations of the cells were imaged by FE–SEM (JSM-7100F, JEOL, Japan) [32].

## 4. Conclusions

The current study reports the green, facile and rapid synthesis of AgNPs using a culture supernatant of *Pseudoduganella eburnea* MAHUQ-39 and also displays their antibacterial efficacy and mechanisms against drug-resistant human pathogens. The method used in this study for green synthesis of AgNPs is rapid, facile, straightforward, convenient, economical and ecofriendly. The strain *Pseudoduganella eburnea* MAHUQ-39 is able to synthesis AgNPs within 24 h of incubation. Moreover, an extracellular method was used to synthesize AgNPs that makes the process easy, rapid and convenient, which could be used for high production. Additionally, the green synthesized AgNPs show significant antimicrobial activity against antibiotic-resistant pathogenic strains of *E. coli*, *S. aureus* and *P. aeruginosa*. MICs of *P. aeruginosa* and *S. aureus* were 6.25 μg/mL and 100 μg/mL, respectively, and the MBCs of *P. aeruginosa* and *S. aureus* were 50 μg/mL and 200 μg/mL, respectively. Synthesized AgNPs can destroy the membrane integrity and cause morphological changes in strains of *P. aeruginosa* and *S. aureus*, leading to cell death. This study may help to describe the antibacterial mechanism of green synthesized AgNPs for both Gram-negative and Gram-positive pathogenic bacteria. This is the first report for the green synthesis of AgNPs using *Pseudoduganella eburnea* MAHUQ-39. The strain *Pseudoduganella eburnea* MAHUQ-39 could be useful for the massive production of AgNPs, and biosynthesized AgNPs can be applied for various applications in medical and nonmedical fields.

## Figures and Tables

**Table 1:** Biochemical characterization of *Pseudoduganella eburnea* MAHUQ-39.

API 20 NE	Result	API ZYM	Result
Nitrate reduction	+	Alkaline phosphatase	+
Indole production	−	Esterase (C4)	+
Glucose fermentation	−	Esterase lipase (C8)	w
**Hydrolysis of**		Lipase (C-14)	w
L-arginine	+	Cystine arylamidase	+
Urea	−	Valine arylamidase	+
Esculine	w	Leucine arylamidase	+
Gelatine	−	Trypsin	+
4-nitrophenyl-BD-galactopyranoside	+	α-chymotrypsin	w
**Utilization of**		Acid phosphatase	+
D-glucose	+	Naphthol-AS-BI-phosphohydrolase	+
D-mannitol	+	α-galactosidase	+
L-arabinose	+	*β*-galactosidase	+
D-mannose	+	*β*-glucuronidase	−
D-maltose	+	*α*-glucosidase	+
*N*-acetyl-glucosamine	+	*β*-glucosidase	+
Gluconate	+	N-acetyl-β-glucosaminidase	+
Adipic acid	−	*α*-mannosidase	w
Capric acid	w	*α*-fucosidase	w
Malic acid	+		
Triosodium citrate	w		
Phenylacetic acid	*+*		

+, Positive; −, Negative; w, weakly positive.

**Table 2:** Antibacterial efficacy of green synthesized AgNPs against *E.coli*, *P. aeruginosa* and *S. aureus*.

Pathogenic Species	Zone of Inhibition (mm)	
	1000 μg/mL	500 μg/mL
*Escherichia coli* [ATCC 10798]	16.1 ± 1.5	13.9 ± 0.8
*Pseudomonas aeruginosa* [ATCC 10145]	16.6 ± 1.3	15.1 ± 1.0
*Staphylococcus aureus* [ATCC 6538]	13.4 ± 0.7	11.6 ± 1.1

**Table 3:** Antibacterial efficacy of commercial antibiotics against *E.coli*, *P. aeruginosa* and *S. aureus*.

Pathogenic Species	Antibiotic	Zone of Inhibition (mm)
*Escherichia coli* [ATCC 10798]	Erythromycin Vancomycin Penicillin G	- - -
*Pseudomonas aeruginosa* [ATCC 10145]	Erythromycin Vancomycin Penicillin G	- - -
*Staphylococcus aureus* [ATCC 6538]	Erythromycin Vancomycin Penicillin G	*-* *-* *-*

## Supplementary Materials

The following are available online at https://www.mdpi.com/1422-0067/21/4/1510/s1, Figure S1: Effect of temperature on the biosynthesis of AgNPs using strain *Pseudoduganella eburnea* MAHUQ-39 was checked on the basis of UV-vis spectral analysis after 24 h of incubation with 1 mM concentration of AgNO_3_, Figure S2: Effect of salt concentration (AgNO_3_) on the biosynthesis of AgNPs using strain *Pseudoduganella eburnea* MAHUQ-39 was checked on the basis of UV-vis spectral analysis after 24 h of incubation at 30 °C.
