# Supplementary material for: Green Synthesis of Silver Nanoparticles Using Pseudoduganella eburnea MAHUQ-39 and Their Antimicrobial Mechanisms Investigation against Drug Resistant Human Pathogens
Source: Int J Mol Sci. 2020 Feb 22;21(4):1510. doi: 10.3390/ijms21041510 (PMC7073201; doi:10.3390/ijms21041510)
Supplement: Supplementary file 1 [file ijms-21-01510-s001.pdf]

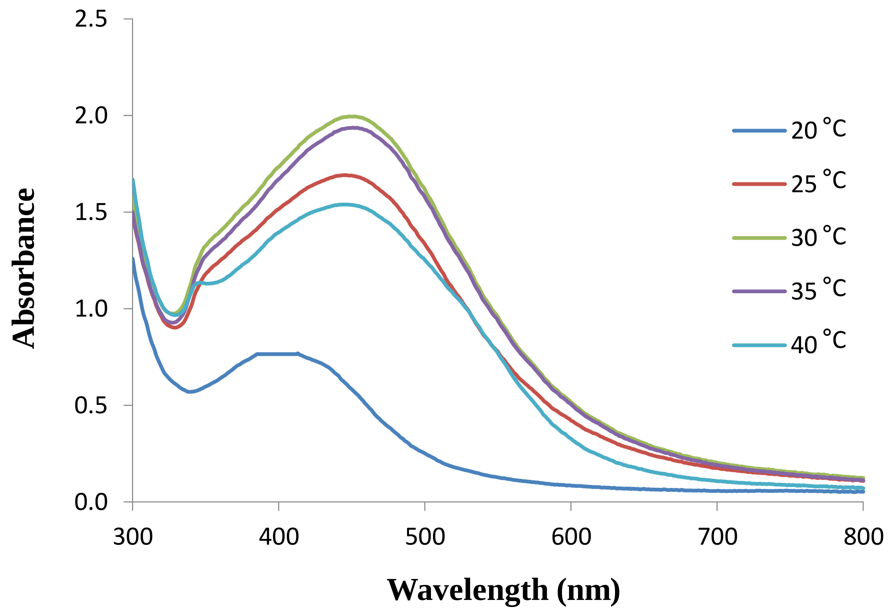

Figure S1. Effect of temperature on the biosynthesis of AgNPs using strain *Pseudoduganella eburnea* MAHUQ-39 was checked on the basis of UV-vis spectral analysis after 24 h of incubation with 1 mM concentration of $AgNO_3$.

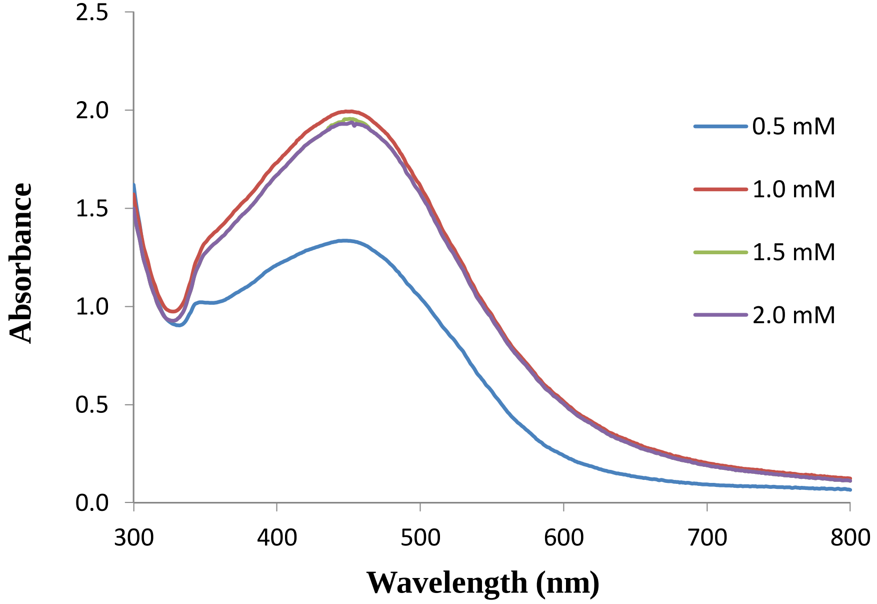

Figure S2. Effect of salt concentration ($AgNO_3$) on the biosynthesis of AgNPs using strain *Pseudoduganella eburnea* MAHUQ-39 was checked on the basis of UV-vis spectral analysis after 24 h of incubation at 30 °C.
